# Comparison Evaluation of the Biological Effects of Sterigmatocystin and Aflatoxin B1 Utilizing SOS-Chromotest and a Novel Zebrafish (*Danio rerio*) Embryo Microinjection Method

**DOI:** 10.3390/toxins14040252

**Published:** 2022-03-31

**Authors:** Zsolt Csenki, Anita Risa, Dorottya Sárkány, Edina Garai, Ildikó Bata-Vidács, Erzsébet Baka, András Szekeres, Mónika Varga, András Ács, Jeffrey Griffitts, Katalin Bakos, Illés Bock, István Szabó, Balázs Kriszt, Béla Urbányi, József Kukolya

**Affiliations:** 1 Department of Environmental Toxicology, Institute of Aquaculture and Environmental Safety, Hungarian University of Agriculture and Life Sciences, 2100 Gödöllő, Hungary; ranita513@gmail.com (A.R.); garaiedina8@gmail.com (E.G.); jeffrey.griffitts@uni-mate.hu (J.G.); csenkibakosk@gmail.com (K.B.); bock.illes@uni-mate.hu (I.B.); szabo.istvan.temi@uni-mate.hu (I.S.); 2 Research Group for Food Biotechnology, Institute of Food Science and Technology, Hungarian University of Agriculture and Life Sciences,1022 Budapest, Hungary; sarkany.dorottya@gmail.com (D.S.); batane.vidacs.ildiko@uni-mate.hu (I.B.-V.); kukolya.jozsef@uni-mate.hu (J.K.); 3 Doctoral School of Biology, Institute of Biology, Eötvös Loránd University, 1117 Budapest, Hungary; 4 Department of Ecotoxicology, Agro-Environmental Research Centre, Institute of Environmental Sciences, Hungarian University of Agriculture and Life Science, 1022 Budapest, Hungary; baka.erzsebet@uni-mate.hu; 5 Department of Microbiology, Faculty of Science and Informatics, University of Szeged, 6726 Szeged, Hungary; szandras@bio.u-szeged.hu (A.S.); varga.j.monika@gmail.com (M.V.); 6 Department of Freshwater Fish Ecology, Institute of Aquaculture and Environmental Safety, Hungarian University of Agriculture and Life Sciences, 2100 Gödöllő, Hungary; acs.andras@uni-mate.hu; 7 Department of Environmental Safety, Institute of Aquaculture and Environmental Safety, Hungarian University of Agriculture and Life Sciences, 2100 Gödöllő, Hungary; kriszt.balazs@uni-mate.hu; 8 Department of Aquaculture, Institute of Aquaculture and Environmental Safety, Hungarian University of Agriculture and Life Sciences, 2100 Gödöllő, Hungary; urbanyi.bela@uni-mate.hu

**Keywords:** mycotoxin, teratogenicity, MAS activation

## Abstract

Aflatoxin B1 (AFB1) is a potent mycotoxin and natural carcinogen. The primary producers of AFB1 are *Aspergillus flavus* and *A. parasiticus.* Sterigmatocystin (STC), another mycotoxin, shares its biosynthetic pathway with aflatoxins. While there are abundant data on the biological effects of AFB1, STC is not well characterised. According to published data, AFB1 is more harmful to biological systems than STC. It has been suggested that STC is about one-tenth as potent a mutagen as AFB1 as measured by the Ames test. In this research, the biological effects of S9 rat liver homogenate-activated and non-activated STC and AFB1 were compared using two different biomonitoring systems, SOS-Chromotest and a recently developed microinjection zebrafish embryo method. When comparing the treatments, activated STC caused the highest mortality and number of DNA strand breaks across all injected volumes. Based on the *E. coli* SOS-Chromotest, the two toxins exerted the same genotoxicities. Moreover, according to the newly developed zebrafish microinjection method, STC appeared more toxic than AFB1. The scarce information correlating AFB1 and STC toxicity suggests that AFB1 is a more potent genotoxin than STC. Our findings contradict this assumption and illustrate the need for more complex biomonitoring systems for mycotoxin risk assessment.

## 1. Introduction

Food and feed contamination by mycotoxin-producing fungi represents a safety risk for the environment, humans, and animals [1] due to the potential mutagenic, carcinogenic, teratogenic, immune-modulating, and cytotoxic effects of their mycotoxins. The most hazardous mycotoxins—the genotoxic aflatoxin B1 (AFB1) and sterigmatocystin (STC)—are produced by members of the *Aspergillus* genus (mostly *Aspergillus flavus*, *A. nidulans*, *A. versicolor*, and *A. creber*). The biosynthetic pathway is shared between AFB1 and STC, since STC serves as the biogenic precursor of AFB1, resulting in very similar polyketide structures but with different biological effects. Based on the harmful biological effects of aflatoxin, the European Union applies the strictest regulatory limits: AFB1 and total aflatoxin derivates should not exceed 2 μg/kg and 4 μg/kg, respectively, in any product intended for direct consumption [2]. The maximum acceptable limit set for aflatoxins in the United States is 20 μg/kg [3]. To date, no country has legislation on permitted levels of STC in food. In Czechoslovakia, before joining the EU, there were STC limits in force in some foods: 50 μg/kg for rice, vegetables, meat, and milk, and 20 μg/kg for other foods [4].

STC was discovered much earlier than AFB1. Studies on antibiotic metabolites of *A. versicolor* reported the isolation of sterigmatocystin in 1948 [5], but these works were not initiated by the recognition of health problems. Sterigmatocystin was first isolated in pure form in 1954 [6], and its structure was completely resolved eight years later [7]. Aflatoxins were discovered in 1961 after cases of acute aflatoxicosis of turkeys (“Turkey × disease”), which resulted in a death toll of approximately 100,000 turkeys [8]. The structure of AFB1 was resolved in 1963 by Asao [9].

AFB1 and STC are turned into mutagenic compounds in both bacterial and mammalian cells after metabolic activation, causing chromosomal damage both in vitro and in vivo in experimental animals [10,11]. These mycotoxins are converted into AFB1 exo-8,9-epoxide and STC 1,2-epoxide, respectively, by cytochrome P450 enzymes. These derivatives can be covalently bound to DNA by forming N7-guanine adducts [12,13]. In a large-scale screening of 300 carcinogenic and non-carcinogenic substances using the standard *S. typhimurium* assay, where the metabolic activation was carried out in the presence of rat liver S9 mixture, STC was approximately 10 times less mutagenic than AFB1 on a molar basis [14]. Baertschi et al. analysed the mutagenicity of metabolically activated STC and AFB1 in a bacterial SOS repair assay using a *S. typhimurium* strain and found that SOS response was slightly higher for STC than for AFB1 [15]. In contrast, Krivobok et al. tested S9-mix-activated STC and AFB1 with the SOS chromotest in *Escherichia coli* PQ37 and showed that AFB1 is the stronger genotoxin [16]. Two different STC metabolites are thought to be responsible for mutagenicity in the literature. The first is highly reactive sterigmatocystin-1,2-epoxide, mentioned above. The other structure was demonstrated by Pfeiffer et al. [17]. In this case, the aromatic ring of the STC is hydroxylated, resulting in a catechol structure. This catechol can react with DNA in a different way to epoxy derivatives. According to Pfeifer’s experiments, less epoxide is produced in human and rat liver microsomes, with catechol being predominantly formed [17].

The genotoxicity of STC is also indicated by the fact that, in rat chromosomes, abnormalities such as chromatid breaks and gaps have been found in the bone marrow cells after 31.2 mg/kg body weight toxin treatment [18]. STC was able to induce DNA double-strand breaks in a human lung cancer cell line (A549 cells). DNA damage has affected key proteins involved in cell cycle regulation, which may be one possible mechanism for the development of lung cancer [19]. In a study by Umeda et al., high concentrations of STC as well as AFB1 showed weak DNA single-strand breaks and chromosomal abnormalities [20]. Given their strong genotoxicity, it is not surprising that both mycotoxins are classified as carcinogenic. Based on animal studies performed to date and cases of human cancer, AFB1 was classified as carcinogenic to humans (Group 1) [21] and STC as possibly carcinogenic to humans (Group 2B) by the International Agency for Research on Cancer [22]. Teratogenic effects of AFB1 include reduced foetal weights, eye socket enlargement, and disturbance of normal heart function in rabbits [23]. In chickens, teratogenic effects, embryonic mortality, and embryonic developmental disorders caused by AFB1 and STC have been reported. [24,25]. To date, STC has been shown to be a teratogenic mycotoxin [11]. Toxicity studies have shown that the oral LD50 range of AFB1 is estimated to be 0.3–17.9 mg/kg body weight in most animal species [26]. Rainbow trout are reported to be one of the most sensitive animal species to AFB1, with an estimated oral LD50 value of 0.5 mg/kg [27]. STC has been shown to be much less toxic in animal studies; the acute toxicity of STC is 10-fold lower than that of AFB1. According to the studies of Butler [28], the LD50 values of STC and AFB1 in rats were 60 and 6 mg/kg b.w., respectively. Of the two mycotoxins, AFB1 has been shown to have a significant immunosuppressive effect, resulting in a reduction in serum total globulin upon treatment [29].

Zebrafish embryos, as a model, are widely used in toxicological and ecotoxicological testing [30,31]. Zebrafish are highly suitable for studying fundamental embryonic development processes, since their embryogenesis is very similar to higher vertebrates, including humans [32,33,34].

In some cases, embryotoxicity is due to metabolites formed during maternal or embryonic metabolism, not the parent compound itself [35]. Bioactivation of parent compounds (proteratogens) may also result in highly toxic metabolites, such as electrophiles or free radical intermediates [36]. Therefore, when testing the proteratogenic potency of compounds with whole embryo models, the addition of a mammalian metabolic activation system (MAS) (e.g., S9-mix, microsomes, or hepatocytes) has been recommended [34,37,38].

Cytochrome P450 enzymes (CYPs) are the most important enzyme families involved in the transformation of xenobiotics, which can result in the toxification of chemicals, e.g., in embryo toxicity [39,40]. Although zebrafish embryos are capable of activating proteratogenic substances without the addition of exogenous metabolic activation systems [41], the presence or absence of CYPs and EHs (Epoxide hydrolases) can hardly be told, as their expression level depends on the developmental stage of the embryo [42,43,44]. Therefore, MAS activation is often used, since it is certain that embryos will be exposed to activated metabolites throughout the treatment.

One disadvantage of MAS activation in zebrafish is the risk of alterations to oxygen saturation values from the organic-rich medium, thereby affecting embryo studies of development [45,46]. To address this issue, we have previously developed a novel microinjection method. Microinjection is a simple way to introduce polar and nonpolar materials as well as organic matter-rich test substances into newly fertilised fish eggs [47,48,49]. With the appropriate settings, the technique enables the administration of exact amounts and ensures the reliability of results [47]. As such, the microinjection of fish embryos could be an alternative method to test materials treated/activated with S9 mix.

The aim of this study was to help address the lack of a complete understanding of STC toxicity. In order to determine the embryotoxic effects of STC compared to AFB1, we used established methods of assessing toxicity in conjunction with a zebrafish model of embryotoxicity which included a novel microinjection method.

## 2. Results

### 2.1. Metabolic Activation

The results of mycotoxin metabolic activation with S9 showed that 71.7% of STC and 72.5% of AFB1 were transformed in the experiment, so the remaining toxin levels were 28.3% and 27.5%, respectively (Table 1; chromatograms are shown in Supplementary Figures S1 and S2). Subsamples showed an almost identical transformation among the three replicate samples taken for the in vivo zebrafish egg toxicity tests.

### 2.2. Genotoxic Potential of AFB1 and STC by SOS Chromotest

**Figure 1:**
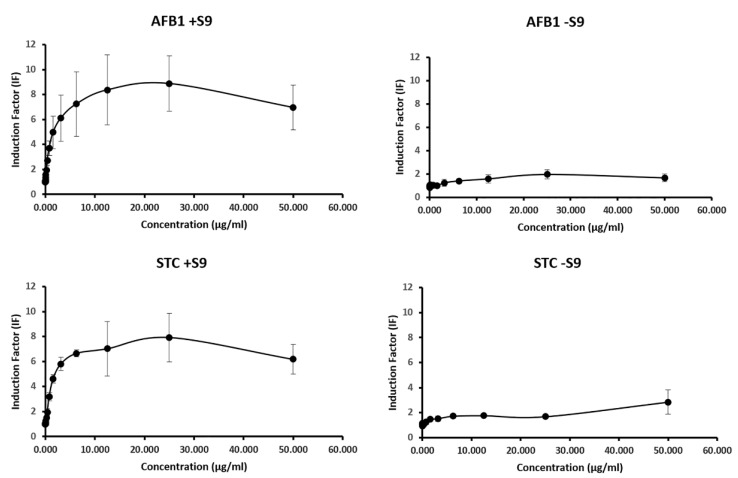
Genotoxic potential of the mycotoxins detected by SOS Chromotest. Normal (−S9) and metabolically activated (+S9) aflatoxin B1 (AFB1) and sterigmatocystin (STC) were examined. (Values represent means ± SD.)

The genotoxicity of normal (−S9) and metabolically activated (+S9) AFB1 and STC was detected by SOS-Chromotest, and the concentration–response curves can be seen in Figure 1.

The non-activated AFB1 (Figure 1 (AFB1 −S9)) showed a negligible increase in induction factors, which means that AFB1 did not have genotoxic activity. Compared to non-activated AFB1, activated AFB1 (Figure 1 (AFB1 +S9)), i.e., the epoxide derivative of the toxin, resulted in a strong increase in SOS response in *E. coli* PQ37 cells, indicating very strong genotoxic potential. In the case of STC without S9 treatment (Figure 1 (STC −S9)), higher induction factors can be observed at higher concentrations than for the non-activated AFB1. According to this finding, STC exhibits weak genotoxicity without metabolic activation. In the case of metabolically activated STC (Figure 1 (STC +S9)), similarly to AFB1, a concentration dependent increase was detected, and the steep increase in induction factors meant strong activation of SOS repair.

SOSIP for genotoxic control 4-nitroquinoline-1-oxide (4NQO) was calculated (SOSIP = 68.51) and a correction factor was determined (CF = 0.96). The dose–response curve for 4NQO is shown in Supplementary Figure S3. The calculated and corrected SOSIP values from the slope of the concentration–response curves enable the comparison of the genotoxicity of the treated and non-treated mycotoxins (Table 2). The calculation of SOSIP values from the slope of the concentration–response curves enables the comparison of the genotoxicity of the treated and non-treated mycotoxins (Table 2). The higher the SOSIP value, the stronger the genotoxic effect of the compound. As observed in Figure 1, AFB1 and STC exhibit remarkable genotoxic effects after metabolic activation (+S9), which is confirmed by the SOSIP calculation. Without metabolic activation, low SOSIP values were obtained in the case of the untreated mycotoxins.

### 2.3. The Effect of Normal and Metabolically Activated Mycotoxins on the Survival of Microinjected Embryos

In this experiment, normal and S9-activated mycotoxins were injected in five different droplet sizes into zebrafish embryos immediately after fertilisation in order to determine mortality effects. In general, it can be stated that as the injected droplet size increased, the proportion of dead individuals also increased for each test substance.

**Figure 2:**
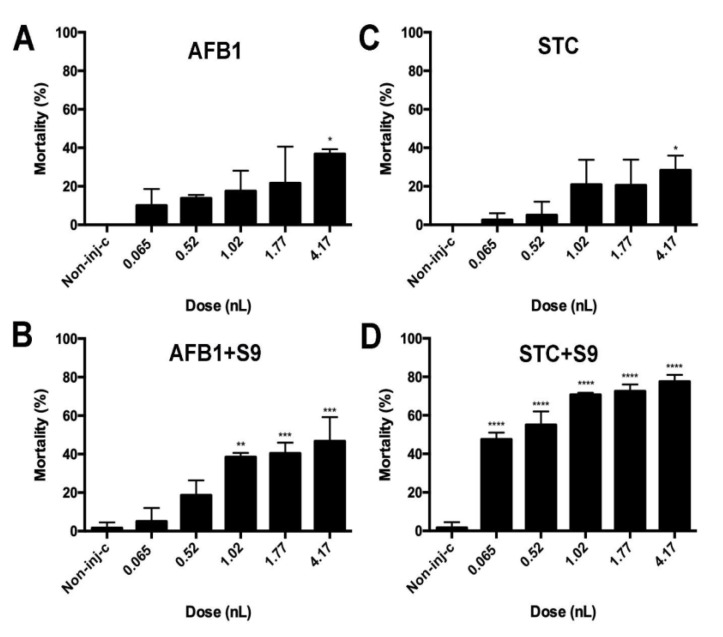
Effects of normal and metabolically activated (+S9) mycotoxins on the survival of the embryos 120 h after treatment. Results were compared to the non-injected control group. (**A**) AFB1. (**B**) AFB1 +S9. (**C**) STC. (**D**) STC +S9. (Values represent means ± SD; * *p* < 0.05; ** *p* < 0.01; *** *p* < 0.001; **** *p* < 0.0001).

Regarding non-activated toxins, a statistical difference was observed only in the case of the largest injected volume (*p* < 0.05) (Figure 2A,C) compared to the non-injected control. Maximum mortality was 36.79% in AFB1, while it was 28.33% in STC.

There was a significant difference (*p* < 0.01) between activated AFB1 and the control group at 1.02, 1.77, and 4.17 nL dosages. However, no statistical difference was observed between normal and activated AFB1 across all injection volumes. The rate of mortality did not reach 100%, even in the case of the largest droplet size (Figure 2B).

In the case of STC +S9 treatment, mortality values exceeded the mortality values of STC at each droplet volume, and the lowest injected volume was significantly different from the control value (*p* < 0.0001). Mortality for the largest injected droplet reached 77.5% (Figure 2D).

Comparing the mortality values of treatments by injected volumes, the STC +S9 treatment was the most toxic of the four investigated samples. The two normal toxins and the activated AFB1 treatment were not statistically different from each other regarding droplet volume (*p* < 0.05) (Table S1).

### 2.4. Sublethal Effects of Normal and Metabolically Activated Mycotoxins in Injected Embryos

**Figure 3:**
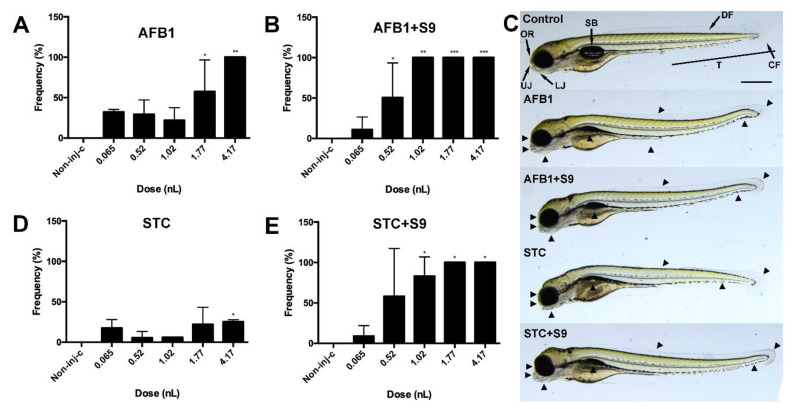
Effects of normal and metabolically activated AFB1 and STC on sublethal effects of embryos 120 h post-treatment (**A**,**B**,**D**,**E**). Results were compared to the non-injected control group. (Values represent mean ± SD; * *p* < 0.05, ** *p* < 0.01, *** *p* < 0.001.) Presentation of representative phenotype lesions (black arrowheads) (**C**). Abbreviations: OR: olfactory region; UJ: upper jaw; LJ: lower jaw; T: tail; CF: caudal fin; DF: dorsal fin; SB: swim bladder. Scale bar: 500 µm.

Sublethal endpoints were also studied on 120 hpf surviving embryos. In general, each treatment of mycotoxin (across all injected volumes) increased the number of deformed embryos compared to the non-injected control group (Figure 3).

Regarding AFB1, a significant difference was seen with the 1.77 nL treatment, while all surviving embryos exhibited sublethal symptoms at all injected volumes. With AFB1 +S9 treatment, the number of deformed embryos was significantly different from the non-injected control group starting at 0.52 nL volume. From 1.02 nL volume, sublethal symptoms reached 100%.

In the case of STC, a significant difference was only found at the largest injected volume, where 17.5% of surviving individuals showed malformation. Activated STC differed significantly from the non-injected control in the appearance of sublethal symptoms beginning at an injection volume of 1.02 nL. Sublethal symptoms affected 100% of the embryos in the two largest volumes.

Analysing the frequency of the sublethal effect of the treatments by injected volumes, it can be stated that there were no significant differences between the treatments with the two smallest volumes. At 1.02 and 1.77 nL, the activated toxins provided a significantly higher rate of distorted embryos than normal toxins. The values of S9-activated toxins were not different from each other. In the largest injected volume, STC resulted in a significantly lower distorted embryo ratio than the other three treatments, which did not differ from each other (Table S2).

Several morphological characteristics indicating developmental dysfunction were observed among all treated animal groups. The most frequently observed morphological abnormalities included: small and not well-defined olfactory regions, moderately bent bodies, mildly wavy dorsal fins, irregular caudal fins, irregularly shaped lower and upper jaws, and uninflated swim bladders (Figure 3C).

### 2.5. Effects of Normal and Metabolically Activated Mycotoxins on DNA Double-Strand Breaks

Genotoxic effects of normal and activated mycotoxins were also investigated on 5-day-old embryos. In general, with the exception of the STC +S9 treatment, none of the test substances changed the number of DNA double-strand breaks significantly compared to the non-injected control (*p* < 0.05).

**Figure 4:**
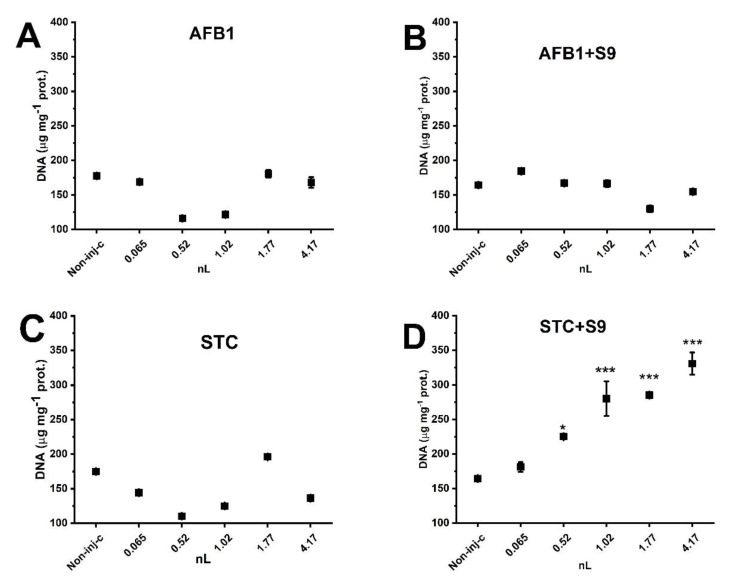
Effects of normal and metabolically activated mycotoxin on DNA fragmentation by 120 h after treatment. Results were compared with those of the non-injected control group. (**A**) AFB1. (**B**) AFB1 +S9. (**C**) STC. (**D**) STC +S9. (Values represent means ± SD; * *p* < 0.05, *** *p* < 0.001.)

There was a slight decrease in DNA strand break values measured for the 0.52 nL and 1.02 nL AFB1 and the 0.52 nL and 1.02 nL STC treatments. Moreover, these values were not significantly (*p* < 0.05) lower compared to the controls. The number of DNA double-strand breaks resulting from activated STC treatment began to increase at a statistically significant level beginning at 0.52 nL injected volume (*p* < 0.05). At the largest activated STC treatment volume, double-strand breaks doubled (330.64 ± 16.07 DNA_sb μg/mg protein), as compared to the non-injected controls (164.39 ± 0.39 DNA_sb μg/mg protein) (Figure 4).

Comparing the results of the four treatment groups, STC +S9 treatment resulted in more DNA double-strand breaks than all others, across all injected volumes (Table S3).

## 3. Discussion

Various studies have aimed to compare the genotoxicity of aflatoxin B1 and sterigmatocystin. Unfortunately, differences, or rather uncertainties, regarding actual mycotoxin concentrations in test organisms and their effects on metabolic pathways and uncertainties about the rate of repair of induced damages make it difficult for a direct comparison between the relative mutagenic potencies of these mycotoxins [51]. For instance, when *Salmonella typhimurium* strains were used in the standard Ames assay, the rat liver S9 mix-activated STC was approximately 10 times less mutagenic than AFB1 [14]. The mutagenicity of STC in *S. typhimurium* strains was confirmed by several studies, but direct comparisons of the results with the effect of AFB1 did not prove consistent. In other studies, STC and AFB1 showed equally strong genotoxicity after activation by human liver enzymes in the Ames test [52], in accordance with the results of Kuczuk and co-workers, who detected nearly identical genotoxicity for these mycotoxins [53]. In the present study, we compared the genotoxicity of AFB1 and STC and their metabolically activated forms at the same concentration and under the same conditions using the SOS Chromotest. For a more accurate comparison, we also determined the induction potential (SOSIP) of the two mycotoxins. Our results showed that activated AFB1 gave SOSIP = 76.96, which is in agreement with published results [50]. Comparative values for STC measured by SOS Chromotest are non-existent. Instead, we compared STC with AFB1 and found that the genotoxicity of STC treated with S9 mix (SOSIP = 75.27) was as strong as that of activated AFB1. Moreover, the two measured SOSIP values did not differ significantly (*p* > 0.05).

In our study, the greatest difference between the biological effects of activated AFB1 and STC was observed in the DNA double-strand break assay, where STC was found to be more aggressive than AFB1. The chromosome damage- and DNA breakage-inducing effects of STC have been well documented in several publications. FM3A cells treated with equal doses (1 μg/mL) of AFB1 and STC were damaged at different scales with STC treatment, resulting in significantly more chromosomal breaks than AFB1 after 24 h incubation [20]. Alkaline comet assays investigating the DNA-damage effect of STC on human neuroblastoma SH-SY5Y cells detected a significant genotoxic increase in DNA damage [54]. It was also shown, using the same assay, that STC caused DNA double-strand breaks in human BEAS-2B and A549 cell lines [19]. Additionally, chromosomal breaks in kidney tissues of Nile tilapia were detected after four weeks of 1.6 μg STE/kg BW STC treatment [55].

An explanation for the difference in the mode of action of the two toxins may be that, in addition to the epoxy derivatives observed during the metabolism of both toxins, STC can be converted to a high percentage of catechol by hydroxylating the aromatic ring, which is also able to react with DNA [17]. This may explain the differences between the behaviour of the two toxins observed in the Ames assays, where AFB1 was found to be ten times more genotoxic than STC, whereas in the SOS-Chromotest they were found to have the same genotoxicity. In the Ames test, guanyl-N7 adducts indicate point mutation capacity and frame shifts, whereas in the SOS-Chromotest DNA breaks can activate the repair system.

Various studies have shown that, in aquatic species, the initial action of AFB1 is similar to that in other species and seems to be highly conserved [56,57]. Many of these fish models have demonstrated high sensitivity, including trout (*p*.o. LD50 0.81 mg/kg bw) and zebrafish (adult LC50 0.58 mg/L; larvae LC50 0.51 mg/L) [58,59]. In contrast, in the case of STC, few studies have investigated the acute effects of the toxin on fish models. The LC50 of STC in adult zebrafish is 0.24 mg/L, which is about half of the AFB1 LC50 [59]. This large difference in LC50 for the two smallest injected volumes can also be seen in our results for non-activated toxins, but for S9-activated toxins, in all cases, a higher toxicity was observed for STC.

AFB1 is teratogenic and showed structural malformation and developmental retardation effects in rodents and rabbits [23,60,61,62,63] and is suspected to lead to reduced birth weight and stillbirth in humans [64]. In zebrafish embryos, deformities of the head, tail, and body axis have been described for AFB1; these were also observed in our study.

The sublethal effects of STC in fish, especially embryos, have not been fully described. Reduced growth rate and observed darker coloration, haemorrhages, and necrosis in the liver and spleen have been reported [55,65,66,67,68]. In our study, we found that STC-induced malformations in zebrafish embryos were the same as in AFB1-treated embryos. The molecular structure of STC is similar to aflatoxins and it acts as an intermediate in the biosynthetic pathway of aflatoxins; therefore, this similarity was expected in the results.

Based on the literature, AFB1 (and probably STC as well) has stage-dependent susceptibility in zebrafish embryos, which is related to the development of the liver and the associated expression of phase I detoxification enzymes, which are primarily localised in the liver [39,40,69,70]. Although initial differentiation of the liver in zebrafish embryos begins at 24 hpf, complete development does not occur until 72–96 hpf [71,72]. Moreover, until approximately 72 hpf, expression of relevant CYP and EH enzymes is low and corresponding xenobiotic-induced activity is not present or is difficult to measure in the embryo, with the expression of these enzymes being primarily linked to liver development [42,43,44]. Nonetheless, zebrafish embryos are able to activate proteratogenic substances without the addition of any exogenous metabolic activation systems and are thus suitable for teratogenicity studies [41]. However, as can be seen from the data in the literature and from our results, there are more pronounced effects with the use of the MAS system.

Microinjection is a simple way to introduce organic matter-rich test substances—such as the MAS-activated samples used in our study—into newly fertilised fish eggs. Microinjection helps to eliminate the risk of hypoxia that could cause a wide range of secondary effects. The method—if optimised well—maintains injection volume variations within ±20% according to the OECD 236 [73] test guideline’s recommendations and provides reliable results [47]. The present study was the first to examine acute toxic effects of S9-bioactivated mycotoxins following microinjection and the results suggest that this technique can be an alternative way to test MAS-activated proteratogenic materials. However, the results of microinjection are difficult to compare with the results of classical tests, where embryos are exposed via waterborne exposure. The microinjection technique enables the administration of exact amounts, so theoretically it would be possible to determine doses per bodyweight as seen in feeding experiments with vertebrates. However, more than one factor in this calculation should be taken into account to accurately determine this value (e.g., absorption rate of the yolk).

## 4. Conclusions

The biological effect of STC is reported as one-tenth that of aflatoxin in the vast majority of studies. According to our results, this also depends on the method or biomonitoring system used by researchers. Using the SOS Chromotest and the zebrafish egg toxicity test, we concluded that STC is a toxin as harmful as AFB1. The great advantage of our newly developed zebrafish egg toxicity test is that it can be used to test extremely small amounts of chemicals or metabolites. It may be possible to separate the metabolic products of STC or AFB1 by preparative HPLC, and after that their biological effects can be tested in order to find the most dangerous metabolic compounds. In the zebrafish egg, the elements of the CYP system are not yet fully activated, making it an ideal system for the toxicological analysis of molecules that are functional after biological activation.

## 5. Materials and Methods

### 5.1. Animal Protection

The Animal Protocol (2013) was approved under the Hungarian Government Regulation on animal experiments (40/2013 (II.4)), and all studies were completed before the treated individuals would have reached the free-feeding stage.

### 5.2. Mycotoxins

AFB1 and STC were purchased from Sigma-Aldrich (Merck, Darmstadt, Germany). Standard solutions were made by diluting the mycotoxin powder with dimethyl-sulfoxide (DMSO, a.r., Reanal Kft., Budapest, Hungary) to make stock solutions of 100 µg/mL. The concentrations of the stock solutions were verified by HPLC measurement.

### 5.3. Metabolic Activation

For metabolic activation of AFB1 and STC, liver homogenates (S9) derived from Aroclor 1254 induced Sprague-Dawley male rats were used [74]. The S9 extract used was part of a SOS Chromotest Kit purchased from Environmental Bio-Detection Products Inc. (Mississauga, ON, Canada). For the activation of enzymes, the S9 mixture contained the following components in addition to the rat liver extract: KCl:MgCl_2_ solution (1.65 M:0.4 M), glucose-6-phosphate solution (0.5 M), nicotine amide di-nucleotide phosphate (NADP) (0.1 M), Tris HCl buffer (0.2 M, pH 7.4), and distilled water.

For zebrafish embryo injection, AFB1 and STC were metabolically activated by S9 liver homogenates in vitro. The metabolic activation was carried out in triplicate for AFB1 and STC in 1 mL volumes (in 1.5 mL microcentrifuge tubes, Eppendorf, Hamburg, Germany); the S9 mix contained the component in equal proportion to the SOS Chromotest. For 1 mL, 5 μg/mL (which corresponds to 16.01 µM AFB1 or 15.42 µM STC) activated mycotoxin solution, 750 μL S9 mix, and 250 μL mycotoxin solution were mixed. The tubes were incubated and shaken at 37 °C for 2 h in a Thermomixer^®^ (Eppendorf, Hamburg, Germany), resulting in metabolically activated AFB1 and STC solutions. The concentration of mycotoxin solutions (0.5 mL) was measured by analytical methods for the controls (without metabolic activation) and the activated mycotoxins.

### 5.4. Mycotoxin Extraction and Analytical Determination

The 0.5 mL control (−S9mix) and metabolically activated AFB1 and STC (+S9mix) samples were lyophilized and stored at −20 °C until extraction. To extract the toxins, 1 mL of methanol/water (6/4) was added to the lyophilized samples followed by sonication at 80 Hz for 3 × 5 min on ice with vortexing of the samples for 1 min between sonication steps. After centrifugation (19,000× *g*, 10 min, 4 °C), the supernatants were subjected to LC-HRMS analysis.

LC-HRMS measurements were performed using an Ultimate 3000 UHPLC system (Thermo Fischer Scientific, Waltham, MA, USA) coupled to a Q Exactive Plus hybrid quadrupole-Orbitrap mass spectrometer (Thermo Fischer Scientific, Waltham, MA, USA) operating with a heated electrospray interface (HESI). AFB1 and STC were separated using a Gemini NX C18 (150 × 2 mm, 3 μm) column (Phenomenex, Torrance, CA, USA) thermostated at 30 °C. Methanol (A) and water (B) mobile phases were supplemented with 0.1% (*v*/*v*%) formic acid. The flow rate was set at 0.2 mL/min, and the injection volume was 5 µL.

All samples were analysed in positive ionisation mode. The mass spectrometer acquired data using a full-scan/data-dependent MS/MS method (Full MS/ddMS2). LC-HRMS data were acquired using Trace Finder 4.0 software (Thermo Fischer Scientific, Waltham, USA). The raw data files were processed by Compound Discoverer™ (2.1) software (Thermo Fischer Scientific, Waltham, USA) for chromatographic alignment, compound detection, and accurate mass identification.

### 5.5. Measurement of the Genotoxic Potential of AFB1 and STC Using the SOS Chromotest Kit

For measurement of the genotoxicity of mycotoxins, an SOS Chromotest Kit (EBPI, Mississauga, ON, Canada) was used according to the manufacturer’s recommendations and formerly optimised guidelines [75]. Tests were performed in 96-well microplates (μClear^®^, Greiner Bio-One Hungary Kft., Mosonmagyaróvár, Hungary), where 10 μL of the tested materials, i.e., AFB1, STC, indirect and direct genotoxic controls, were added to the microplate and two-fold serial dilutions were carried out twice using 10% dimethyl sulfoxide (DMSO) in 0.85% saline as a dilution. The AFB1 and STC were tested in the range of 50–0.001 μg/mL. Solutions of 2-Amino-Anthracene (2AA), an indirect genotoxin, and 4-Nitro-Quinoline-Oxide (4NQO), a direct genotoxin, were used as positive controls, and 10% DMSO in saline was applied as a negative control. The optical density of the overnight bacterial culture was adjusted to OD_600_ = 0.05. One serial dilution of each compound and 2AA received the bacterial suspension containing S9 mix for metabolic activation, and 100 μL bacterial suspensions without S9 mix was added to the second serial dilution of the compounds and 4NQO. The microplate was incubated at 37 °C for 2 h in a microplate incubator (Biosan PST-60HL-4 Plate Shaker-Thermostat, Latvia). Subsequently, 100 μL of substrate mix (containing 10 mL X-gal and 50 mg p-nitrophenyl-phosphate) was added to the wells. The plate was further incubated for 1.5 h in the dark. After blue and yellow colours developed, the absorbance was measured by an ELISA reader (ELx800 Absorbance Reader, BioTek Instruments, Winooski, VT, USA) at 405 and 620 nm wavelengths. The test was carried out in three replicates. 

Induction factor (IF) was calculated from the absorbance values according to Equation (1) [76]:

Induction factor (IF) = (*C*405 × *S*620)/(*S*405 × *C*620) (1)

where *C* is the mean of the absorbance values of the control and *S* is the mean of the absorbance values of the sample measured at 405 and 620 nm wavelengths. Concentration–response curves derived from IF points measured at different concentrations of the mycotoxins and controls indicated the SOS inducing potency (SOSIP) [77].

### 5.6. Zebrafish Maintenance and Egg Collection

Laboratory-bred AB strain zebrafish were held in breeding groups of 30 females and 30 males at the Institute of Aquaculture and Environmental Safety, Hungarian University of Agriculture and Life Sciences, Hungary, in a Tecniplast ZebTEC recirculation system (Tecniplast S.p.A., Buguggiate, Italy) at 25.5 ± 0.5 °C, pH 7.0 ± 0.2, conductivity 550 ± 50 µS (system water), and light:dark period of 14 h:10 h. Fish were fed twice a day with dry granule food (Zebrafeed 400–600 µm, Sparos Lda., Olhão, Portugal) supplemented with freshly hatched live *Artemia salina* twice a week. Fish were placed in breeding tanks (Tecniplast S.p.A.) late in the afternoon the day before the experiment and allowed to spawn by removing the dividing walls the next morning. Spawning of individual pairs was delayed through time to allow a continuous supply of 1-cell embryos.

### 5.7. Zebrafish Embryo Microinjection

Microinjection was conducted as described by Csenki et al. [47] with only a few adjustments in the volumes injected: 0.074 nL (droplet diameter 50 µm) corresponding to 1 × 10^–9^ µM AFB1 or 1 × 10^–9^ µM STC, 0.22 nL (droplet diameter 75 µm) corresponding to 8 × 10^–9^ µM AFB1 or 8 × 10^–9^ µM STC, 1.02 nL (droplet diameter 125 µm) corresponding to 1.6 × 10^–8^ µM AFB1 or 1.6 × 10^–8^ µM STC, 1.77 nL (droplet diameter 150 µm) corresponding to 2.8 × 10^–8^ µM AFB1 or 2.7 × 10^–8^ µM STC, 4.17 nL (droplet diameter 200 µm) corresponding to 6.7 × 10^–8^ µM AFB1 or 6.4 × 10^–8^ µM STC.

In each treatment, 20 eggs were injected, at a minimum, three times. Eggs were then incubated in the system water with methylene blue (2 mL 0.1% methylene blue in 1 L system water) (25 ± 2 °C) in 10 cm diameter Petri dishes. Developing embryos were selected and transferred in groups of twenty into 6 cm diameter Petri dishes following 2 h of incubation. Coagulated and non-fertilised eggs were discarded. Petri dishes were then incubated at 26 ± 1 °C under a 14 h:10 h light:dark cycle. Embryos were examined for lethal and sublethal abnormalities under a microscope. System water was renewed daily until 120 hpf (hours post fertilisation).

### 5.8. Examination of Injected Embryos

Embryo mortality was determined on the basis of egg coagulation, the lack of somite formation, and the lack of heart function at 120 hpf. Sublethal endpoints (pericardial edema, yolk edema, tail deformation, craniofacial deformation, and disintegrated abnormal embryo shape) were also determined at 120 hpf. Abnormalities were examined separately, irrespective of the number of deformities in the individual. Digital images were captured of laterally oriented 120 hpf larvae at 30× magnification using a stereomicroscope (Leica M205 FA, Leica DFC 7000T camera, Leica Application Suite 3.4.2.18368, Leica Microsystems GmbH, Wetzlar, Germany). 

### 5.9. Sample Preparation for DNA Damage Measurement

To quantify DNA damage, 120 hpf larvae were pooled into 2 mL Eppendorf centrifuge tubes and stored at −80 °C until measurement. Fish larvae were homogenised using a small bead mill (TissueLyser LT, Qiagen, Germantown, MD, USA) in a general buffer (25 mM Hepes-NaOH, 130 mM NaCl, 1 mM EDTA, 1 mM dithiothreitol, pH 7.4) at a weight-to-volume ratio of 1:5. DNA strand breaks were quantified according to the alkaline precipitation assay described by Olive adapted to microplate [78]. Tissue homogenates (25 μL) were mixed, then incubated at 60 °C for 10 min with 200 μL of 2% SDS (containing 10 mM EDTA, 10 mM Tris-base, and 40 mM NaOH) and 100 μL of 0.12 M KCl. Then, the solution was cooled to 4 °C for 30 min and centrifuged at 8000× *g* for 5 min at 4 °C. A subsample of the supernatant (50 μL) was added to 150 μL of Hoechst dye in a 96-well microplate (1 μg/mL, in 0.1 M Tris-acetate buffer, pH 8.5–9, containing 0.4 M NaCl, 4 mM sodium cholate) and mixed for 5 min in a plate reader. Fluorescence was measured at 360 excitation/450 nm emission wavelengths using a multimode plate reader (Thermo Varioskan LUX). In control blanks, the tissue homogenate was replaced by 50 μL Hepes buffer. A salmon sperm DNA standard (Sigma) was used for DNA calibration. The protein content of the raw homogenates was determined in triplicate by the Bradford method [79], adapted to microplate, using bovine serum albumin as a standard. The absorbance was recorded at 595 nm after an incubation period of 15 min. The results were expressed as DNA_sb μg/mg protein.

### 5.10. Statistics

Results were analysed and graphs were plotted using GraphPad Prism 6.01 (GraphPad Software, San Diego, CA, USA). Normality checks on the data were performed with the Shapiro–Wilk normality test and non-compliance with parametric methods was established. Significant differences were verified by Kruskal–Wallis analysis with Dunn’s multiple comparisons test.

## Figures and Tables

**Table 1:** Aflatoxin B1 (AFB1) and sterigmatocystin (STC) concentration values of normal (−S9) and metabolically activated (+S9) samples used in the fish embryotic injection experiments. (Values represent means ± SD).

Samples	Mean Toxin Concentration (µg/mL)
STC −S9	5.16 ± 0.16
AFB1 −S9	5.20 ± 0.18
STC +S9	1.46 ± 0.24
AFB1 +S9	1.43 ± 0.23

**Table 2:** SOS inducing potency (SOSIP) of aflatoxin B1 and sterigmatocystin detected by SOS Chromotest. Results were compared to the data reported in the scientific literature. (Values represent means ± SD.)

	SOSIPin Our Results		SOSIPin the Literature		Reference
	+S9	−S9	+S9	−S9	
AFB1	76.96 (±4.91)	1.28 (±0.45)	75.0	n.d.	[50]
STC	75.27 (±7.54)	1,13 (±0.48)	n.d.	n.d.	-

## Data Availability

We have full control of all primary data, and we agree to allow the journal to review our data if requested.

## Supplementary Materials

The following supporting information can be downloaded at: www.mdpi.com/article/10.3390/toxins14040252/s1, Table S1: Effect of normal and metabolically activated (+S9) mycotoxin on the survival of the embryos 120 h post treatment. Different letters indicate significant differences (*p* < 0.05), Table S2: Effect of normal and metabolically activated (+S9) mycotoxin on the appearance of distorted embryos compared to all surviving embryos by 120 h post treatment. Different letters indicate significant differences (*p* < 0.05), Table S3: Effect of normal and metabolically activated (+S9) mycotoxin on DNA fragmentation compared to 120 h post treatment. Different letters indicate significant differences (*p* < 0.05). Figure S1: Extracted ion chromatogram of AFB1 eluted at Rt = 20.7 min in the AFB1 −S9 (A) and FB1 +S9 (B) samples as well as the representative MS/MS mass spectrum of AFB1 (C) recorded in the eluted peak; Figure S2: Extracted ion chromatogram of STC eluted at Rt = 28.2 min in the STC −S9 (A) and STC +S9 (B) samples as well as the representative MS/MS mass spectrum of STC (C) recorded in the eluted peak; Figure S3: Dose-response curves of positive control 4-nitroquinoline-1-oxide (4NQO) used in SOS-Chromo test for determination of the correction factor. (Values represent mean ± SD.).
