# Supplementary material for: Comparison Evaluation of the Biological Effects of Sterigmatocystin and Aflatoxin B1 Utilizing SOS-Chromotest and a Novel Zebrafish (Danio rerio) Embryo Microinjection Method
Source: Toxins (Basel). 2022 Mar 31;14(4):252. doi: 10.3390/toxins14040252 (PMC9027791; doi:10.3390/toxins14040252)
Supplement: Supplementary file 1 [file toxins-14-00252-s001.zip › toxins-1624155-suppl-update.pdf]

Supplementary Materials: Comparison Evaluation of the Biological Effects of Sterigmatocystin and Aflatoxin B1 Utilizing SOS-Chromotest and a Novel Zebrafish (*Danio rerio*) Embryo Microinjection Method

Zsolt Csenki1, Anita Risa, Dorottya Sárkány, Edina Garai, Ildikó Bata-Vidács, Erzsébet Baka, András Szekeres, Mónika Varga, András Ács, Jeffrey Griffitts, Katalin Bakos, Illés Bock, István Szabó, Balázs Kriszt, Béla Urbányi, József Kukolya

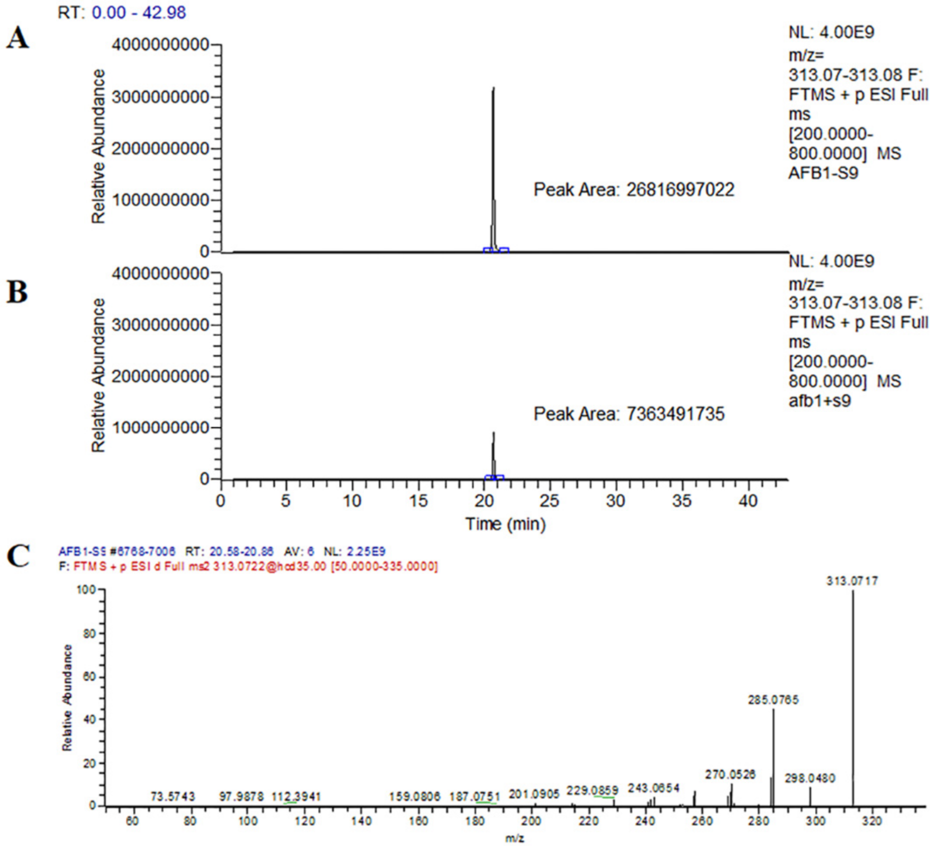

Figure S1. Extracted ion chromatogram of AFB1 eluted at Rt = 20.7 min in the AFB1-S9 (**A**) and FB1+S9 (**B**) samples as well as the representative MS/MS mass spectrum of AFB1 (**C**) recorded in the eluted peak.

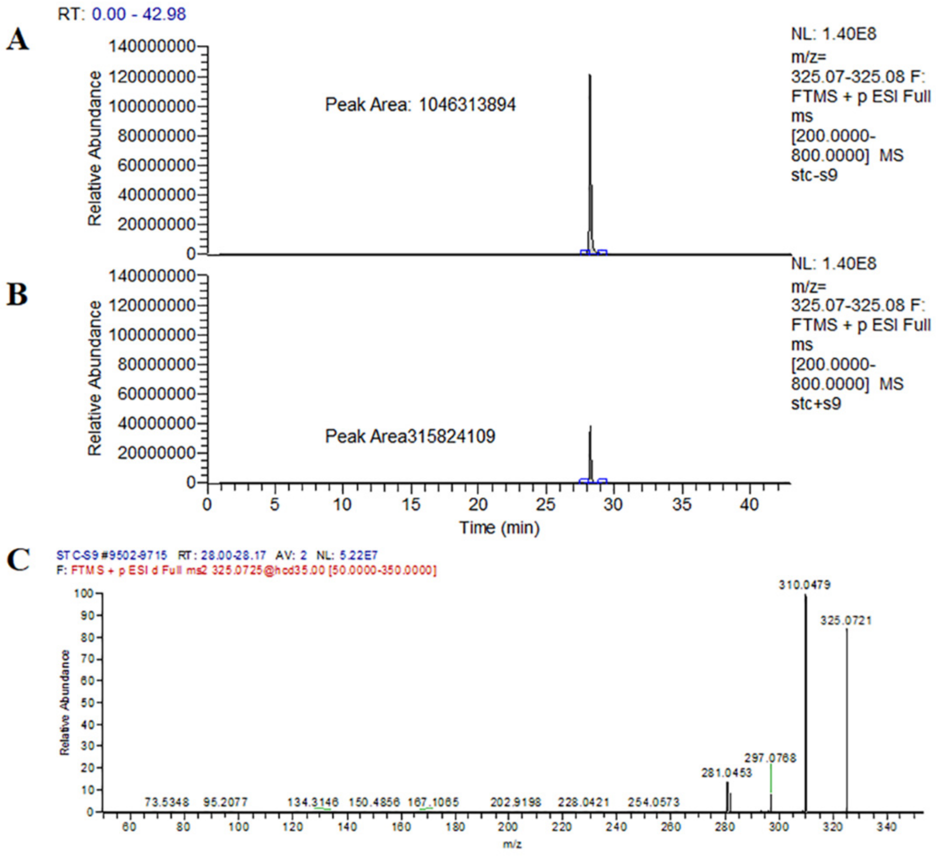

Figure S2. Extracted ion chromatogram of STC eluted at Rt = 28.2 min in theSTC-S9 (**A**) and STC+S9 (**B**) samples as well as the representative MS/MS mass spectrum of STC (**C**) recorded in the eluted peak.

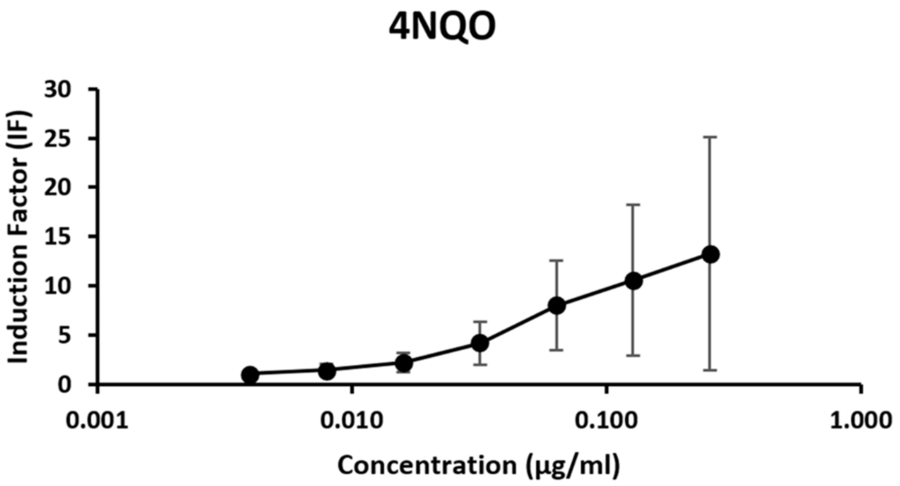

Figure S3. Dose-response curves of positive control 4-nitroquinoline-1-oxide (4NQO) used in SOS-Chromo test for determination of the correction factor. (Values represent mean ± SD.).

Table S1. Effect of normal and metabolically activated (+S9) mycotoxin on survival of the embryos by 120 hours post-treatment. Different letters indicate significant differences (p <0.05). (Values represent mean ± SD.).

	Mortality (%)			
	**AFB1**	**AFB1+S9**	**STC**	**STC+S9**
0.074 nL	10.00^{a} (±8.66)	5.00^{a} (±7.07)	2.50^{a} (±3.54)	47.50^{b} (±3.54)
0.52 nL	13.75^{a} (±1.77)	18.61^{a} (±7.74)	5.00^{a} (±7.07)	55.00^{b} (±7.07)
1.02 nL	17.50^{a} (±10.61)	38.42^{a} (±2.23)	20.88^{a} (±12.90)	70.72^{b} (±1.01)
1.77 nL	21.67^{a} (±18.93)	40.38^{a} (±5.58)	20.56^{a} (±13.36)	72.50^{b} (±3.54)
4.17 nL	36.79^{a} (±2.53)	46.67^{a} (±12.58)	28.33^{a} (±7.64)	77.50^{b} (±3.54)

Table S2. Effect of normal and metabolically activated (+S9) mycotoxin on the appearance of distorted embryos compared to all surviving embryos by 120 hours post-treatment. Different letters indicate significant differences (p <0.05). (Values represent mean ± SD.).

	Deformed (%)			
	**AFB1**	**AFB1+S9**	**STC**	**STC+S9**
0.074 nL	32.22 (±3.06)	11.00 (±15.56)	17.50 (±10.61)	9.09 (±12.86)
0.52 nL	29.50 (±17.68)	50.67 (±42.74)	5.50 (±7.78)	58.34 (±58.92)
1.02 nL	22.00^{a} (±15.56)	100.00^{b} (±0.00)	6.00^{a} (±0.00)	83.34^{b} (±23.92)
1.77 nL	57.65^{a} (±39.07)	100.00^{b} (±0.00)	22.00^{a} (±15.00)	100.00^{b} (±0.00)
4.17 nL	100.00^{a} (±0.00)	100.00^{a} (±0.00)	25.33^{b} (±1.45)	100.00^{a} (±0.00)

Table S3. Effect of normal and metabolically activated (+S9) mycotoxin on DNA fragmentation compared to 120 hours post-treatment. Different letters indicate significant differences (p <0.05). (Values represent mean ± SD.).

	DNA double strain break			
	**AFB1**	**AFB1+S9**	**STC**	**STC+S9**
0.074 nL	168.94^{a} (±1.85)	184.52^{ab} (±3.27)	144.30^{ab} (±1.69)	181.16^{b} (±7.05)
0.52 nL	116.23^{a} (±0.72)	167.14^{b} (±0.59)	110.22^{a} (±0.58)	225.04^{c} (±2.59)
1.02 nL	121.69^{a} (±0.47)	166.46^{b} (±4.66)	124.95^{a} (±2.96)	280.00^{c} (±24.95)
1.77 nL	180.55^{a} (±5.41)	130.07^{b} (±4.85)	196.23^{a} (±2.26)	285.29^{c} (±3.14)
4.17 nL	168.06^{a} (±7.68)	154.71^{a} (±2.92)	136.43^{a} (±0.66)	330.64^{b} (±16.07)
